# Expression and clinical significance of MCF2L-AS1 in stomach adenocarcinoma

**DOI:** 10.1016/j.clinsp.2025.100701

**Published:** 2025-07-10

**Authors:** Chaowen Sun, Jun Guo, Bixia Chen, Xiayang Lu, Huihui Jiang

**Affiliations:** a Department of Gastrointestinal and Hernia Surgery, Guilin People’s Hospital, China; b Department of Oncology, Northern Jiangsu People’s Hospital, China; c Department of Gastroenterology, Jiangmen Central Hospital, China; d Department of Gastroenterology, Taizhou Traditional Chinese Medicine Hospital, China; e Department of Spleen and Stomach Diseases, Yichang Hospital of Traditional Chinese Medicine, China

**Keywords:** MCF2L-AS1, miR-503–5p, STAD, Prognosis, Cellular processes

## Abstract

**Background:**

Stomach Adenocarcinoma (STAD) poses a significant burden due to its high prevalence and costly, painful treatments, exerting considerable pressure on individuals.

**Objectives:**

This study intends to explore novel therapeutic targets to enhance prognosis and alleviate patient stress.

**Materials and methods:**

Quantitative Real-time Polymerase Chain Reaction was employed to detect MCF2L-AS1 expression in STAD tissues and cell lines. The correlation between this expression and patients' clinical conditions and prognosis was analyzed utilizing the Chi-squared test and Kaplan-Meier method. To investigate the regulatory mechanism of MCF2L-AS1, the Luciferase reporter gene system and transfection experiments were implemented. Cellular behaviors were analyzed through CCK8 and Transwell assays.

**Results:**

MCF2L-AS1 expression was upregulated in STAD tissues and cells, strongly correlating with TNM stage and lymph node metastases. High MCF2L-AS1 levels were associated with reduced 5-year survival rates compared to the low-expression groups. miR-503–5p, a downstream miRNA, was downregulated in STAD and inversely correlated with MCF2L-AS1. Knockdown of MCF2L-AS1 suppressed miR-503–5p expression, indicating its role as a competitive endogenous RNA. Low miR-503–5p expression reversed the inhibitory effects of MCF2L-AS1 knockdown on STAD cell behaviors.

**Conclusions:**

The oncogene role of MCF2L-AS1 in STAD is mediated through the negative regulation of miR-503–5p, highlighting its potential as a prognostic marker and therapeutic target.

## Introduction

Gastric cancer incidence continues to rise globally, posing a substantial threat to public health.^1^^,^^2^ As the predominant histological subtype, Stomach Adenocarcinoma (STAD) is the main type of gastric malignant tumor. Currently, most patients relapse and gradually progress to advanced disease after tumor resection, with only about 30 % five-year survival rate post-recurrence. Although advanced STAD patients can receive chemical and radiation therapy, these treatments' efficacy is limited due to potentially severe side effects. The prognosis for STAD patients remains poor, causing significant economic and emotional burdens on patients' families.^3^ Therefore, extending the lifespan of STAD patients and improving their quality of life pose serious challenges for doctors and scientific researchers.

Non-coding RNAs (ncRNAs)are functionally defined by their inability to encode proteins. Based on their length, they are divided into two groups: long ncRNAs (lncRNAs, more than 200 nucleotides) and small ncRNAs (sncRNAs, less than 200 nucleotides).^4^^,^^5^ Several studies have demonstrated that lncRNAs can regulate the occurrence and progression of STAD, acting as potential oncogenes or tumor suppressor genes r.^6^ In STAD, the upregulated expression of LINC00152 predicted metastasis and deterioration, promoting cancer cells through the ERK/MAPK signaling pathway.^7^ Similarly, upregulated HOTAIR promotes the expression of HER2 by negatively regulating miR-331–3p/miR-124, leading to a worsened STAD phenotype and shortened patient survival time.^8^ Conversely, PTENP1 was significantly downregulated in STAD tissues, and when overexpressed, it inhibited the proliferation and promoted the apoptosis of STAD cells.^9^ Another tumor suppressor gene, lncRNA Loc490, is negatively associated with lymph node metastasis in STAD and positively associated with overall survival.^10^

Zheng et al. discovered that MCF2L-AS1 is integral to specific tumor-associated ceRNA networks in gastric cancer, highlighting its pivotal role in tumor initiation and progression.^11^ In colorectal cancer, MCF2L-AS1 fosters disease progression by sponging MiR-105–5p, which targets to the Ras oncogene family protein rabb-22a (RAB22A).^12^ Further research into the immune landscape of lung adenocarcinoma revealed a correlation between MCF2L-AS1 and macrophage infiltration.^13^ In non-small cell lung cancer, high expression of MCF2L-AS1 accelerates cancer progression through negative modulating miR-873–5p^14^ However, the function of MCF2L-AS1 in STAD remains elusive. This study endeavors to be the first to elucidate the role and underlying mechanism of MCF2L-AS1 in STAD, enhancing the comprehension of its potential regulatory pathways.

The aim of this study was to preliminarily investigate the expression of MCF2L-AS1 in STAD tissues and cells, to analyze the correlation between its expression level and clinicopathological features of the patients, and to further reveal the function of MCF2L-AS1 and its molecular mechanism of action in STAD cell lines, with a view to providing new perspectives and rationale for advancing the research of gastric adenocarcinoma

## Materials and methods

### Source of tissue samples

In this investigation, 117 tissue and nearby normal tissue samples were collected during the surgical procedure at STAD, which is from the Department of General Surgery, Guilin People’s Hospital (approval number: 202,000,198). The Medical Ethics Committee at Guilin People’s Hospital has granted official approval for this study, and all specimens were obtained with patients' complete informed consent.

### Inclusion criteria for patients

To ensure that the tissue specimens used meet the design requirements of this project, the tissue samples must also meet the following standards:

1) Inclusion criteria:
  a) Primary STAD diagnosed by more than 2 senior pathologists.
  b) No history of gastric and esophageal surgery.
  c) The patient corresponding to the specimen had not received any adjuvant chemoradiotherapy or targeted therapy before surgery.
  d) The patient did not undergo Endoscopic Submucosal Dissection (ESD).
  e) Patients informed and consented to specimen collection.
2) Exclusion criteria:
  a) Gastric cancer specimens diagnosed as non-STAD, such as tumors of other gastric histological types, tumors of neighboring organs invading gastric tissue, or metastatic cancers of organ origin other than the stomach.
  b) Received any adjuvant chemoradiotherapy, targeted therapy, ESD surgery before surgery.
  c) Patients with a history of gastric and esophageal surgery and/or recurrence after surgery.

### Cell culture

The cell lines were obtained from Biochemical and Cellular Biology Institute (IBCB, China). They include GES-1 (human gastric mucosa epithelial cells) and a series of STAD cell lines: AGS, HGC-27, MKN-45, and SNU-16. These cells were stored in liquid nitrogen and were thawed and recovered before use. The revived cells were then placed into DMEM medium (Thermo Fisher Scientific, Waltham, USA) with 10 % Fetal Bovine Serum (FBS). They were transferred to a 37 °C, 5 % CO_2_ incubator for incubation. During this period, cell conditions were closely monitored, and medium exchanges were performed timely based on cell growth and nutrient depletion. Upon achieving approximately 80 % confluency within the culture dish, a cell passaging procedure is initiated to maintain cellular health and avoid overcrowding.

### Cell transfection

The aforementioned cultured cells were collected and grouped, then placed in serum-free DMEM medium. After resuspension, the cells were seeded 12-well culture plates and incubated in a stable environment until reaching a cell density of 60 %‒80 %. At this point, the medium was replaced with serum- containing DMEM medium prior to transfection. During transfection, Lipofectamine 2000 (Invitrogen Life Technologies, Carlsbad, CA) was mixed with the transfection sequences: si-MCF2L-AS1 and its negative control (si-NC), si-MCF2L+miR-503–5p inhibitor (si+miR-inhibitor), and its corresponding negative control (si+miR-NC), to form transfection complexes. These complexes were then transferred to a stable environment for further incubation for 48 hours.

### Quantitative real-time polymerase chain reaction (QRT-PCR)

Adhering to the protocol of Trizol reagent (Tiangen, Beijing, China)), total RNA was extracted from tissues and cells. Subsequently, RNA samples with an A260 nm/A280 nm ratio ranging from 1.9∼2.1 were selected using the NanoDrop 1000 UV–Vis Spectrophotometer (Thermo Fisher Scientific, Wilmington, USA). Following the instructions provided by the reverse transcription kit (TaKaRa, Dalina, Japan), the selected total RNA samples were reverse transcribed into cDNA. The cDNA was further amplified according to the protocol of the SYBRRT-RT-qPCR Mixture (TaKaRa, Dalina, Japan). During data analysis, the amplification conditions were as follows: 95 °C (10 min), 25 cycles, 95 °C (15 min), 60 °C (60 min). The primer sequences were designed by Shanghai Biological Engineering Co Ltd (Table S1). GAPDH and U6 were chosen as reliable housekeeping genes, and the relative expression levels of target genes under different conditions were calculated by the 2^-ΔΔct^ method 2.4 CCK8 assay.

The cells required for the experiment were collected and digested using trypsin to prepare single-cell suspensions. After digestion, they neutralized the trypsin with serum-containing medium and centrifuge to remove the enzyme. The cells were resuspended in fresh medium and seed them into 96-well plates at a predetermined density. The plates were gently shaken to ensure even cell distribution, then incubated them in a 37 °C, 5 % CO_2_ incubator. According to the experimental design, cell viability assays were performed at 0-, 24-, 48-, and 72-hours post-seeding. Prior to the assay, researchers prepared a CCK-8 working solution by mixing CCK-8 reagent with serum-free medium in a designated ratio (following the manufacturer's instructions), taking care to protect it from light. At each time point, the appropriate volume of the CCK-8 working solution was added to each well. *R* The plates were returned to the incubator for an additional 4 hours of incubation. Finally, the Optical Density (OD_450_) of each well was measured at 450 nm using a microplate reader.

### Transwell assay

When performing the Transwell experiment, serum-free cell suspensions were prepared first. For migration assays, the prepared cell suspension was directly introduced into the upper chamber of the Transwell plate. Concurrently, the lower chamber of the Transwell was filled with serum-containing medium. Subsequently, the plate was incubated in a 37 °C, 5 % CO_2_ humidified incubator for 24 hours. Upon completion of incubation, the Transwell membrane was meticulously rinsed to eliminate non-migrated cells and medium residues. Thereafter, the migrated cells were fixed with an appropriate methanol solution and visualized through crystal violet staining. In invasion assays, a layer of Matrigel is pre-coated onto the upper chamber surface to mimic the in vivo extracellular matrix environment. The remaining steps were the same as those of the migration experiment.

### Dual-luciferase reporter assay

Potential binding sites for MCF2L-AS1 and miR-503–5p in the 3′UTR region were predicted using the LncRNASNP v3 website (http://gong_lab.hzau.edu.cn/lncRNASNP3#!/). Wild-type and mutant 3′UTR luciferase reporter gene plasmids for MCF2L-AS1 (WT-MCF2L-AS1 and MT-MCF2L-AS1) were constructed using dual luciferase reporter plasmids (pGL3-basic, GenePharma, Shanghai, China). And they were co-transfected into cancer cells with miR-503–5p mimic, miR-503–5p inhibitor, and miR-NC, respectively. The Lipofectamine® 2000 double luciferase reporter assay kit was utilized to detect the MCF2L-AS1 luciferase activity 48 hours post-transfection.

### Statistical analysis

Statistical analyses were conducted using SPSS 23.0, and results were visualized using GraphPad Prism 7.0. All tests were independently replicated three times, and the measurement data were presented as mean ± standard deviation. Differences between two groups were analyzed using the student’s *t*-test, while differences among multiple groups were evaluated through ANOVA with subsequent post-hoc tests. Categorical data were analyzed for associations using the Chi-Squared (χ^2^) test. The Cox proportional hazards model was used for prognostic assessment to explore influencing factors, and the Kaplan-Meier curves were employed to visually depict survival outcomes. Statistical significance was set at *p* < 0.05, ensuring the scientific validity and effectiveness of the conclusions.

## Results

### Expression and clinical significance of MCF2L-AS1 in STAD

**Fig. 1:**
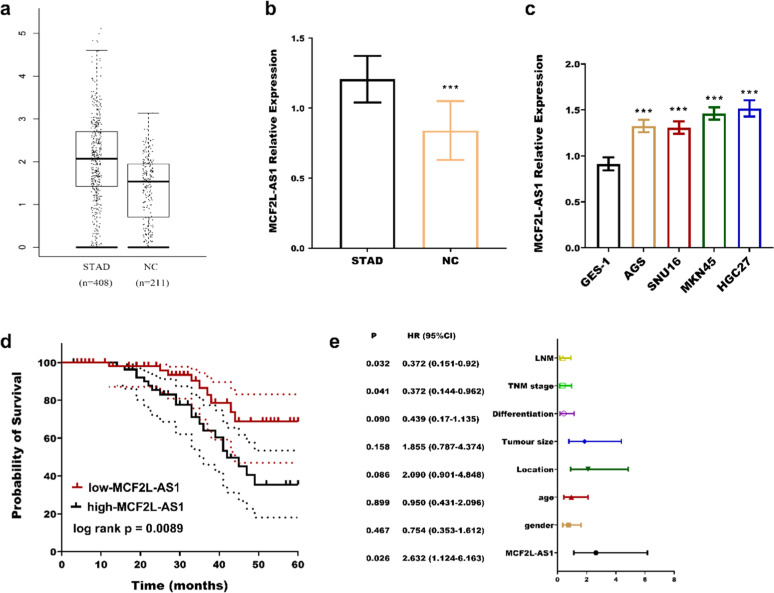
Elevated MCF2L-AS1 expression in STAD correlates with disease progression and poor prognosis. MCF2L-AS1 expression was upregulated in STAD from the GEPIA (a) database, and the study (b) compared to normal tissues. MCF2-AS1 expression was higher in STAD cells than in normal cells (c). High MCF2L-AS1 expression is associated with poor patient prognosis (d). MCF2L-AS1 is a risk factor for poor outcomes with STAD cancer (e). ***p < 0.001, compared with normal tissues and normal cells. LNM, Lymph Node Metastases.

From the GEPIA dataset (http://gepia.cancer-pku.cn/index.html), it was found that MCF2L-AS1 was upregulated in STAD (Fig. 1a). In this experiment, MCF2L-AS1 was significantly upregulated in STAD tissues (Fig. 1b) and four cancer cells (Fig. 1c). According to the average expression level of MCF2L-AS1, 117 STAD patients were divided into two groups, namely the low expression group (63) and high expression group (54) and found that the two groups had notable differences in TNM stage (*p* = 0.023) and lymph node metastasis (*p* = 0.013) (Table 1). In addition, the low expression group had a better prognosis compared with the high MCF2L-AS1 expression group (log rank *p* = 0.0089, Fig. 1d). TNM stage (*p* = 0.041), Lymph node metastasis (*p* = 0.032), and MCF2L-AS1 (*p* = 0.026) can be used as independent biological indicators of patient outcome (Fig. 1e).

**Table 1:** The Association of MCF2L-AS1 with patients’ clinicopathological features.

Variant	Cases (*n* = 117)	MCF2L-AS1 expression		p
		Low (*n* = 58)	High (*n* = 59)	
Gender				0.929
Male	61	30	31	
Female	56	28	28	
Age				0.781
< 45	59	30	29	
≥ 45	58	28	30	
Location				0.16
Proximal	65	36	29	
Middle/Distal	52	22	30	
Tumor size (cm)				0.301
≤ 5	65	35	30	
> 5	52	23	29	
Differentiation				0.069
Well-moderate	71	40	31	
Poor	46	18	28	
TNM stage				0.023
I‒II	77	44	33	
III	40	14	26	
Lymph node metastasis				0.013
No	78	45	33	
Yes	39	13	26	

### Function and molecular mechanism of MCF2L-AS1 in STAD

**Fig. 2:**
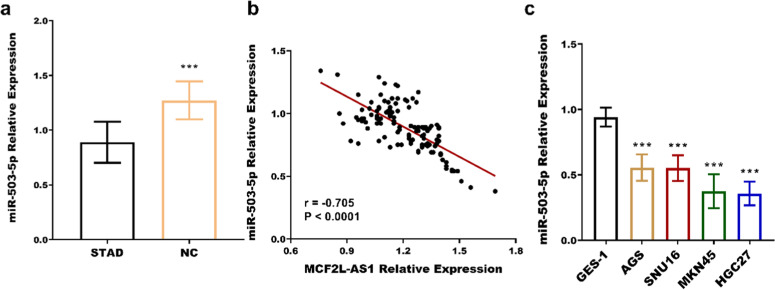
miR-503–5p expression is elevated in STAD and negatively correlated with MCF2L-AS1. miR-503–5p was significantly upregulated in STAD compared with normal tissues (b) and cells (c). miR-503–5p was negatively correlated with MCF2L-AS1 (c). ***p < 0.001, compared with normal tissues and normal cells.

**Fig. 3:**
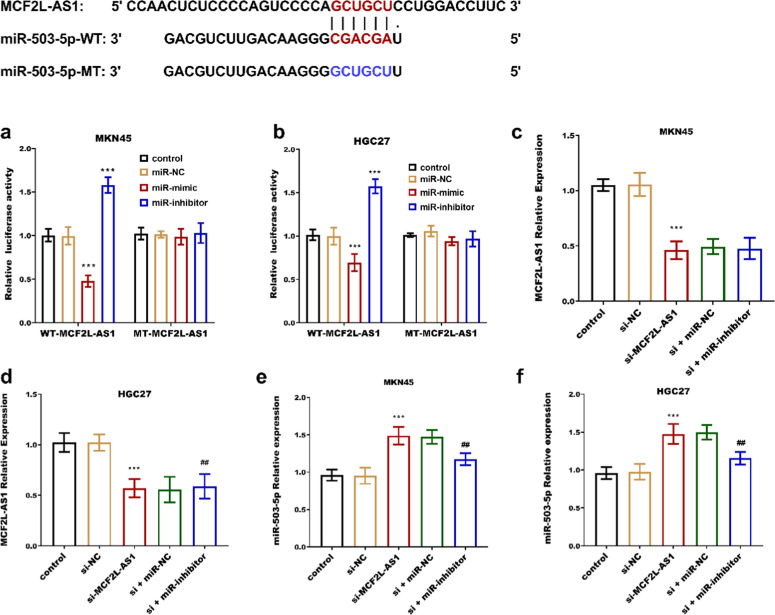
miR-503–5p targets MCF2L-AS1. miR-503–5p overexpression decreased the activity of MCF2L-AS1 luciferase (a‒b). Transfection of si-MCF2L-AS1 knocked down MCF2L-AS1 expression (c‒d) and promoted miR-503–5p expression (e‒f). *** *p* < 0.001, compared with control group; ^##^*p* < 0.01 compared with the si-MCF2L-AS1 group.

**Fig. 4:**
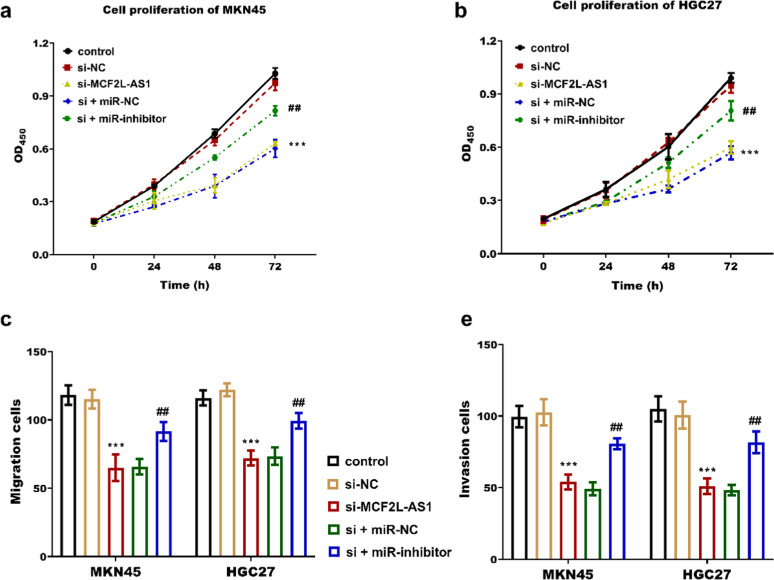
MCF2L-AS1 enhances STAD cell behaviors, counteracted by miR-503–5p silencing. MCF2L-AS1 promotes the proliferation (a‒b), migration (c) and invasion (d) of STAD cells, while silencing miR-503–5p reverses the inhibitory effect of MCF2L-AS1 knockdown. ** *p* < 0.01, compared with control group; ^##^*p* < 0.01 compared with the si-MCF2L-AS1 group.

Further exploration of the mechanism of MCF2L-AS1 in STAD revealed that miR-503–5p is a downstream miRNA of MCF2L-AS1. The miR-503–5p was significantly downregulated in STAD tissues (Fig. 2a) and four cancer cells (Fig. 2b), contrary to the pattern of MCF2L-AS1 expression. Association analysis of the two revealed that miR-503–5p was negatively associated with MCF2L-AS1 (*r* = −0.705, *p* < 0.0001, Fig. 2c). The luciferase activity of MCF2L-AS1 was reduced when miR-503–5p was overexpressed, and vice versa (Fig. 3a‒b). MCF2L-AS1 expression was lowered by si-MCF2L-AS1 (Fig. 3c‒d), while miR-503–5p expression was upregulated (Fig. 3e‒f). The negative impact of MCF2L-AS1 knockdown on the malignant behavior including proliferation (Fig. 4a‒b), migration (Fig. 4c) and invasion (Fig. 4d) of STAD cells was reversed by miR-503–5p downregulation. It indicated that MCF2L-AS1 promoted the activity of STAD cells by targeting miR-503–5p

## Discussion

The continuous development of tumor therapies, especially the application of targeted drugs, has significantly improved patient prognoses. However, the overall effect of targeted drug therapy for STAD remains unsatisfactory.^15^ Therefore, there is an urgent clinical need to identify sensitive genes closely related to the occurrence and development of STAD, providing targets for gene-targeted therapy. Dysregulation of lncRNAs has been linked to STAD progression, for example, LncRNA GAPLINC can modulate CD44-dependent cell invasion, correlating with poor prognosis in STAD patients.^16^ Additionally, LncRNA GHET1 promotes STAD proliferation by enhancing C-Myc mRNA stability.^17^ Consequently, this research aims to discover novel lncRNAs as specific biomarkers, establishing a foundation for improving the prognosis of STAD patients.

Recent studies have highlighted the oncogenic role of MCF2L-AS1 across multiple cancer types. In breast cancer, elevated MCF2L-AS1 expression predicts shortened patient survival,^18^ while in colorectal cancer, it drives tumor progression by sponging miR-105–5p to promote RAB22A-mediated metastasis.^12^ Expanding on these findings, the present study systematically investigated the clinical significance of MCF2L-AS1 in STAD. The authors observed that MCF2L-AS1 is markedly upregulated in both STAD tissues and cell lines, with its high expression strongly correlating with advanced TNM stages, lymph node metastasis, and reduced patient survival rates. This expression pattern aligns with Zheng et al.'s research in gastric cancer^11^ and expands the clinical value of MCF2L-AS1 ‒ its expression level can serve as an independent prognostic marker.

Malignant cell behaviors are closely linked to cancer progression and prognosis. Accumulating evidence suggests that MCF2L-AS1 regulates these processes in a tissue-specific manner. For example, in liver cancer, it enhances malignant phenotypes through the miR-33a-5p/ELAVL1 axis.^19^ Functional analyses in the present study revealed that knocking down MCF2L-AS1 significantly inhibits the proliferation, migration, and invasion of STAD cells. Notably, despite its conserved oncogenic function, MCF2L-AS1 exhibits tissue-specific regulatory networks. For example, in colorectal cancer, it activates miR-105–5p, which regulates the resistance of cancer cells to oxaliplatin,^20^ whereas it promotes invasiveness by adsorbing miR-874–3p^21^ This heterogeneity underscores the necessity of identifying cancer-specific downstream targets of MCF2L-AS1 to unravel its precise mechanisms in STAD.

The competing endogenous RNA (ceRNA) hypothesis posits that lncRNAs regulate gene expression by competitively binding to miRNAs, thereby liberating miRNA-targeted mRNAs.^22^^,^^23^ It was found that miR-503–5p was the downstream miRNA of MCF2L-AS1. miR-503–5p binds to VEGF-A in colorectal cancer, thereby inhibiting the progression of cancer cells.^24^ In osteosarcoma, the miR-503–5p/CDCA4 axis regulates the genesis and development of cancer cells.^25^ This comprehensive study has elucidated that miR-503–5p exerts a regulatory influence on the luciferase activity of MCF2L-AS1. Specifically, the targeted suppression of MCF2L-AS1 leads to an upregulation of miR-503–5p, subsequently impeding the proliferation of cancer cells. Conversely, the downregulation of miR-503–5p reverses this inhibitory effect, indicating a functional interplay between the two. Consequently, it is hypothesized that MCF2L-AS1 may facilitate the progression of STAD by modulating miR-503–5p levels, thereby providing novel insights into the molecular pathways governing this disease.

While this study establishes a functional link between MCF2L-AS1 and STAD progression via the miR-503–5p axis, it has limitations, including single-center sample size and the absence of in vivo validation using animal models. Additionally, the specific downstream mRNA targets of miR-503–5p in STAD remain undefined, leaving the complete regulatory network of MCF2L-AS1 partially uncharacterized. Future studies could address these gaps by expanding sample cohorts, performing mechanistic analyses in xenograft models, and identifying direct miR-503–5p target genes to fully elucidate the MCF2L-AS1/miR-503–5p axis in STAD. Such efforts would not only strengthen the translational potential of MCF2L-AS1 as a prognostic biomarker but also pave the way for developing targeted therapies against this lncRNA-driven oncogenic pathway.

In conclusion, the upregulation of MCF2L-AS1 emerges as a potential harbinger of STAD progression, metastasis, and adverse prognosis. Furthermore, it actively participates in oncogenesis by inhibiting the function of miR-503–5p, thereby underscoring its pivotal role in the advancement of STAD. This observation contributes to a deeper understanding of the molecular mechanisms underlying the disease.

## Availability of data

The datasets used and/or analyzed during the current study are available from the corresponding author upon reasonable request.

## Funding

No funding was received to assist with the preparation of this work.

## CRediT authorship contribution statement

**Chaowen Sun:** Conceptualization, Formal analysis, Software, Validation, Writing – original draft, Writing – review & editing. **Jun Guo:** Conceptualization, Investigation, Software, Validation, Writing – original draft, Writing – review & editing. **Bixia Chen:** Data curation, Project administration, Supervision, Visualization, Writing – original draft, Writing – review & editing. **Xiayang Lu:** Data curation, Resources, Supervision, Visualization, Writing – original draft, Writing – review & editing. **Huihui Jiang:** Conceptualization, Methodology, Software, Visualization, Writing – original draft, Writing – review & editing.

## Conflicts of interest

The authors declare no conflicts of interest.

## Appendix Supplementary materials

Image, application 1
