# Supplementary material for: Expression and clinical significance of MCF2L-AS1 in stomach adenocarcinoma
Source: Clinics (Sao Paulo). 2025 Jul 10;80:100701. doi: 10.1016/j.clinsp.2025.100701 (PMC12275226; doi:10.1016/j.clinsp.2025.100701)
Supplement: Supplementary file 1 [file mmc1.docx]

**CLINICS-D-25-00218_Supplementary Material**

**Table S1** Primer sequences.

| **Primer name** | | **Primer sequence** |
| --- | --- | --- |
| MCF2L-AS1 | Forward | 5'-GATCAACGTTCAATCCACCG-3' |
|  | Reverse | 5'-CGTCAAGATAGCGCAGCTTCC-3' |
| GAPDH | Forward | 5'-CTCTGCTCCTCCTGTTCGAC-3' |
|  | Reverse | 5'-GCGCCCAATACGACCAAATC-3' |
| miR-503-5p | Forward | 5'-CCTATTTCCCATGATTCCTTCATA-3' |
|  | Reverse | 5'-CTCGTTCGGCAGCACA-3' |
| U6 | Forward | 5'-AACGCTTCACGAATTTGCGT-3' |
|  | Reverse | 5'-CTCGTTCGGCAGCACA-3' |
